# Military Career Adaptability Questionnaire in China: Development and Validation

**DOI:** 10.3389/fpsyg.2020.00280

**Published:** 2020-03-10

**Authors:** Wenmo Zhang, Yuanyuan Xu, Li Peng, Chen Bian, Yongju Yu, Ying Li, Min Li

**Affiliations:** ^1^Department of Social Medicine and Health Service Management, Army Medical University (Third Military Medical University), Chongqing, China; ^2^Department of Fundamental, Army Logistical University, Chongqing, China; ^3^Department of Military Psychology, College of Psychology, Army Medical University, Chongqing, China; ^4^School of Sociology and Law, Sichuan International Studies University, Chongqing, China

**Keywords:** career adaptability, Chinese military personnel, validity, reliability, psychometrics

## Abstract

**Objective:**

To develop the Military Career Adaptability Questionnaire (MCAQ) in China and to test its reliability and validity.

**Methods:**

In study 1, an open-ended questionnaire survey was conducted among 200 military personnel. Based on the empirical construction by military personnel of various branches, the dimensions of the MCAQ were constructed, and a preliminary questionnaire was prepared. In study 2, the questionnaire survey was conducted in 1,578 participants enrolled through stratified cluster sampling. They were randomly divided into two groups (*n* = 789). Sample 1 was used for item analysis and exploratory factor analysis, and sample 2 was used for confirmatory factor analysis and internal consistency testing. In sample 1, the participants were selected to test the test–retest reliability of the questionnaire at a 4 weeks interval. In sample 2, participants were selected to test criterion validity using the Psychological Capital Questionnaire and the Job Satisfaction Questionnaire.

**Results:**

According to study 1, we obtained an initial 23-item MCAQ containing five dimensions (organization and fusion ability, communication ability, learning development ability, emotion regulation ability, and career transformation ability). After the exploratory factor analysis in study 2, 21 items contributing 72.17% of the total variance remained. Via the subsequent confirmatory factor analysis, the model was confirmed to have good fit indices [chi-square/degree of freedom (*X*^2^/*df*) = 3.11, goodness of fit index (GFI) = 0.90, normed fit index (NFI) = 0.91, incremental fit index (IFI) = 0.94, tucker lewis index (TLI) = 0.93, comparative fit index (CFI) = 0.94, standardized root mean square residual (SRMR) = 0.07]. These five factors were significantly correlated with the total score of the MCAQ (*r* = 0.73–0.79, *p* < 0.01). The Cronbach α coefficient of the questionnaire was 0.92; the Cronbach α coefficients of the five factors were 0.89, 0.83, 0.88, 0.84, and 0.79, respectively, the test–retest reliability of the questionnaire was 0.93.

**Conclusion:**

The MCAQ developed in this study has a clear five-factor structure and good reliability and validity. It can be used to assess the career adaptability of military personnel to provide a theoretical basis for military vocational psychological education.

## Introduction

Currently, the rapid information technology development, high-tech device application, demanding tasks, and the extreme environment of the battlefield are all challenging for the professional psychological status of military personnel (Bates et al., 2010; Hu et al., 2018), not to mention the problems caused by equipment renewal, job rotation, and residency relocation (Zhao et al., 2018), all posing challenges for the professional psychological status of military personnel. Therefore, every Chinese soldier of the new era should actively develop the ability to adapt to the needs of the military and the professional environment based on his or her own inner self, i.e., the career adaptability of military personnel in the new era. The career adaptability of military personnel not only reflects the ideological and psychological conditions of soldiers in daily life but also affects their cognitive capability and behavior on the battlefield. Therefore, it is necessary to conduct a study on the career adaptability of military personnel in the new era.

Career adaptability, advocated by Savickas, an American psychologist, is a hot topic in the field of professional psychology. Currently, strengthening employees’ career adaptability consciously will be helpful to their occupational success (Xu et al., 2017). Career adaptability has a significant impact on career development and work- and life-related variables. For example, career adaptability can promote the successful career transition of individuals (Brown et al., 2012; Guan et al., 2015b); positively predicts performance, with this relationship partially mediated by career self-management (Gao et al., 2019); and improve individuals’ satisfaction with their work and life (Zacher and Hannes, 2014; Xie et al., 2016), as well as their well-being (Maggiori et al., 2013; Hartung and Taber, 2008) and job performance. It is serves as an important predictor for indicators of well-being, such as life satisfaction (Rudolph et al., 2017); it is positively correlated with the academic achievement of adolescents (Negru-Subtirica and Pop, 2016); it is related to the job content plateau (Jiang, 2016); it can reduce the anxiety, feelings of failure (Pouyaud et al., 2012), and work stress (Nilforooshan and Salimi, 2016) of individuals; and it plays an intermediary role between proactive personality and internal professional growth (Hu and Cheng, 2017). Therefore, career adaptability is also regarded as “the individual’s psychological adaptability in dealing with tasks, problems, turning points and even major events in the career” and as “the key ability or meta-ability of individuals for professional achievement” (Hartung et al., 2011). Furthermore, the majority of published studies on career adaptability include the perspectives of college students and employees. However, compared to these groups, military personnel have to deal with a multifarious range of high-intensity training activities, a fully enclosed environment, and highly consistent organizational management in their career, and they must take into account the specific organizational structure and mission of the military.

The career adaptability of military personnel refers to the special ability of individuals to adapt to changes in the military environment and to solve actual problems in their own career. It is the ability of individuals to adapt to military operations and to face and solve professional problems. Military career adaptability is not a general concept referring to psychological resources or quality but the special ability required to meet professional requirements as a member of the military. Different from other measurement models of career adaptability, our model for military personnel has two features: (1) its theory is reality-oriented rather than future-oriented, and (2) the constructs are based on “capability” rather than “psychological resources.”

Thus, what are the dimensions of military career adaptability? How do we assess it? This is a major concern for researchers. Regarding the constructs and measurement of career adaptability, first, the well-recognized theory is the four-factor career adaptability construct proposed by Savickas: career focus, career control, career curiosity, and career self-confidence (Savickas and Porfeli, 2012). However, the emphasis on self-concept and identity development in career development is in line with the individualism of Western countries, which may not be consistent with the development of Eastern culture, which has a stronger sense of collectivism (Guan et al., 2015a). According to Savickas, career adaptability is a future-oriented psychosocial resource that an individual needs to have in anticipation of and to plan for his or her future career, and in preparation for what might come next (Savickas and Porfeli, 2012). Thus, the adaptable individual is conceptualized as becoming concerned about his or her vocational future, taking control of trying to prepare for one’s vocational future, and displaying curiosity by exploring possible selves and future scenarios. However, unlike most other careers, military personnel are confronted with unpredictable changes in their career life all the time. Therefore, there are few options in their future career development, which is determined by the development of the organization instead. In the army, the adaptability of an individual does not mean being concerned about the future or curious about future scenarios. Their adaptability may not be explained in Savickas’ way. It should focus on the special ability to adapt to military operations and face the changes of their current occupation, rather than being dependent on concern, control, curiosity, and confidence. It is more reality-oriented rather than future-oriented. Second, Chinese scholar Zhao Xiaoyun carried out an exploratory study on the career adaptability of college students and proposed six factors influencing the career adaptability of Chinese college students: career control, career curiosity, career concern, career self-confidence, career adjustment, and career interpersonal relations. He also developed a career adaptability questionnaire (Zhao et al., 2015) that can feasibly predict the future career adaptability of college students. However, since military personnel have few career options compared to college students, some mental resources may not be suitable for measuring the career adaptability of contemporary Chinese military personnel. Third, in 2014, with qualitative research and related research methods, Wang Yifu developed a five-dimensional theory of the career adaptability of enterprise employees. He determined that their career adaptability was characterized by an “external construct” and a “reality orientation” (Wang, 2014). Wang’s career adaptability scale has good reliability and validity; however, it, too, is not applicable to contemporary military personnel, because of the specificity of military occupations.

Due to the difference between Eastern and Western culture and the difference between military organizational culture and enterprise culture, the career development path of military personnel and the future career development path of students are different. Based on existing career adaptability theories and constructs of experience, we developed a concept and dimensions of military career psychological adaptability through a qualitative study, compiled the Military Career Adaptability Questionnaire (MCAQ) in China, and conducted a large-sample test on the reliability and validity of the MCAQ, to apply the theory of military career adaptability to clinical psychology, to improve the mental health and work performance of military officers and soldiers, and to elevate their fighting capacity.

## Study 1

The aim of study 1 was to generate a pool of items that comprehensively structure and measure the dimensions of military career adaptability through qualitative research and to examine the content of the items.

### Methods

#### Participants

An open-ended questionnaire survey was conducted among 200 military personnel of different ranks, educational backgrounds, and lengths of military service from the navy, army, and air force as well as rocket troops and joint logistics forces (127 males and 73 females; 78 participants from the army, 62 participants from the navy, 35 participants from the air force, and 25 participants from other branches; 145 participants with a bachelor’s degree or above). Institutional ethical approval and informed consent were obtained for all studies reported in this article. The questionnaire was administered to 200 participants, and 185 valid questionnaires were collected for preliminary item construction.

An expert panel was recruited to assess the content and face validity of the generated items. The expert panel consisted of eight academics (i.e., four professors in psychology, two experts with doctoral degrees, and two experts with master’s degrees).

#### Procedure

Qualitative research was carried out by using open-ended questionnaires.

(a) According to the results of the questionnaires, representative phrases and words were extracted for classification, and the response items were summarized. (b) Then, themes were further abstracted, and the empirical structure of the MCAQ was established. (c) Following initial item generation, the content and face validity of the items were examined by the expert panel. The two experts with doctoral degrees in psychology reverse-categorized the items that had been summarized and compared them with the results obtained by the researchers; the two experts with master’s degrees in psychology were informed of several subject categories, and after discussion, they categorized the summarized items into the corresponding themes to verify the reliability of the content analysis.

The experts worked independently of each other and were provided with the opportunity to make any suggestions that they felt might help improve each item or to suggest new items. The four professors were informed of the context and structure of the questionnaire and the instructions for examining the content validity of the items.

#### Measures

Qualitative research data were collected using open-ended questionnaires (Wang, 2014). The main issues included the following: (a) Since your enlistment, what kinds of problems that you have encountered have required adaptation? (b) What capabilities should a soldier have for adaptation? (c) In your opinion, what factors will affect the career adaptability of military personnel? (d) If you have excellent career adaptability, what are the positive effects that you have experienced? If you have poor career adaptability, what are the adverse effects?

#### Analysis and Results

A total of 120 items were summarized under “major problems in military career development,” which contains five topics (military skills and learning development, relationships and family, the military operating environment and pressure, organizational atmosphere and management, and career transition and adaptation). A total of 79 items were summarized under the “ability to work out career development issues in the military,” which consists of a five-dimensional military career adaptability construct: organization and fusion ability, communication ability, learning development ability, emotion regulation ability, and career transformation ability. Based on the results of the qualitative research, the items with high cumulative frequency were selected as the measurement variables or indicators. After review and revision by the four psychology professors, a preliminary career adaptability questionnaire for military personnel was developed. At this stage, items were retained if (a) the cumulative frequency of the items was significantly high; (b) there was 80% agreement that the items loaded onto the same construct; and (c) the items were rated as being highly representative of the content of the construct (M > 3/4).

All the experts independently reviewed and revised the appropriateness of the dimension construction and item selection of the questionnaire, with the result that they all agreed with the dimensional structure of the questionnaire. The experts suggested revisions to some of the items, which were revised in the formal test. The initial version of the MCAQ was developed. A total of 23 measurement variables were determined. The communication ability dimension includes five variables: interpersonal relationships, comrade-in-arms communication, superior and subordinate relationships, loneliness, and family communication. The organization and fusion ability dimension includes six variables: management system and norms, management style, professional honor, spirit of sacrifice, teamwork, and perseverance. The learning development ability dimension includes four variables: professional knowledge, learning ability, self-knowledge, and command ability. The emotion regulation ability dimension includes four variables: work stress, environmental change, work frustration, and closed environment. The career transformation ability dimension includes four variables: job transfer, secondary employment, career expectations, and emergencies. All measurement variables are in a single-item response mode, and one measurement variable corresponds to one question. The questionnaire is a self-report instrument and is scored using a seven-point Likert scale. The total score for the career adaptability of military personnel is equal to the sum of the scores for each dimension. The higher the total score, the stronger the career adaptability.

## Study 2

The aim of study 2 was to test the initial factor structure of the MCAQ (consisting of 23 items) generated in study 1 through exploratory factor analysis (EFA) and confirmatory factor analysis (CFA); the reliability and validity of the MCAQ were also tested.

### Methods

#### Participants and Procedure

The formal questionnaire survey was conducted among 1,578 participants enrolled though stratified cluster sampling, and 1,264 valid questionnaires were collected (80.10%). They were randomly divided into two groups (*n* = 789). A preliminary questionnaire (sample 1) was used for item analysis and EFA. A total of 610 valid questionnaires were collected from sample 1 (77.31%). A formal questionnaire (Sample 2) was used for CFA and internal consistency reliability testing. A total of 654 valid questionnaires were collected from sample 2 (82.89%). The basic situation is shown in Table 1. In sample 1, 450 valid participants from a regiment of the army were selected to test the test–retest reliability of the questionnaire 4 weeks later. A total of 474 valid participants from sample 2 were randomly selected to test the criterion validity of the questionnaire.

**TABLE 1:** Descriptive statistics for all samples (*N* = 1,264).

		Sample 1 (*N* = 610)	(%)	Sample 2 (*N* = 654)	(%)
Gender	Male	531	87.0	544	83.2
	Female	79	13.0	110	16.8
Category	Soldier	72	11.8	60	9.2
	Petty office	324	53.1	313	47.8
	Officer	214	35.1	281	43.0
Only children	Yes	236	38.7	243	37.2
Married	Yes	167	27.4	223	34.1
Education	Doctor	11	1.8	12	1.8
	Master	190	31.1	291	44.5
	Bachelor and below	409	67.1	351	53.7
Army corps	Ground force	285	46.7	297	45.4
	Navy	158	25.9	183	28.0
	Sky force	20	3.3	34	5.2
	Rocket force	147	24.1	140	21.4
Army life	More than 30 years	8	1.3	11	1.7
	20–30 years	68	11.1	21	3.2
	10–20 years	118	19.4	123	18.8
	5–10 years	147	24.1	182	27.8
	1–5 years	269	44.1	317	48.5

All evaluators were professionals with a bachelor’s degree or above in psychology and systemic training. The survey was held on site, with standardized instructions and a unified testing environment. All participants signed an informed consent form. The valid questionnaires recovered were randomly and equally divided into two groups: one for CFA on the structural model of the questionnaire and the internal consistency reliability test and the other for testing criterion-related validity. Finally, a standard career adaptability questionnaire for military personnel that conformed to psychometric criteria was developed.

#### Measures

##### Military career adaptability

This variable was measured using five items from the MCAQ. The MCAQ adopted a seven-point Likert scale ranging from 1 to 7 for 21 self-reported items. The MCAQ included five dimensions: organization and fusion ability, communication ability, learning and development ability, emotion regulation ability, and career transformation ability. The internal consistency coefficient of the MCAQ was 0.92.

##### Psychological capital

The Psychological Capital Questionnaire (PCQ) adopted a six-point Likert scale ranging from 1 to 6 for 24 self-reported items. The PCQ included four dimensions, i.e., self-efficacy, hope, optimism, and resilience, referring to mental states that can be measured, developed, and managed (Luthans and Youssef, 2004). Luthans et al. (2004) argued that psychological capital refers to the state of mind that can produce active behaviors of individuals. Psychological capital helps improve the job satisfaction of military personnel and reduce the tendency to leave, seriously affecting the morale and fighting capacity of troops, and it is regarded as an important factor in the career adaptability of military personnel. The internal consistency coefficient of the PCQ was 0.93.

##### Job satisfaction

The Job Satisfaction Questionnaire (JSQ) adopted a six-point Likert scale ranging from 1 to 6 for six self-reported items. The internal consistency coefficient for the JSQ was 0.84. It is assumed that the job satisfaction of military personnel is positively correlated with their career adaptability.

#### Data Analysis

Using SPSS 19.0 software, item analysis, correlation analysis, and EFA were performed for the data from sample 1, and internal consistency reliability testing was performed for the data from sample 2. First, item-total statistics were used to test whether all items were consistent with the scale. Inconsistent items were removed based on the results. Second, the Kaiser–Meyer–Olkin (KMO) test and Bartlett’s test of sphericity were used to test whether the data were appropriate for factor analysis. Third, a series of principal component analyses (PCAs) was used to explore the latent structure of the MCAQ item set. The criteria for dimensions and item reduction (Worthington and Whittaker, 2006) were as follows: (1) eigenvalues greater than 1; (2) factors containing three or more items; (3) items loading strongly (>0.30) onto factors; and (4) items not cross-loading onto two or more factors. A total of 474 participants in sample 2 were selected to test the criterion validity of the questionnaire. For the sample 2 data, AMOS 23.0 software was used for CFA.

## Results

### EFA

#### Item Analysis

First, according to the total score on the questionnaire, all participants were divided into two groups: a high-score group (27% of all participants) and a low-score group. The differences in each item between the high-score and low-score groups were analyzed. The results showed that all items presented statistically significant differences between the two groups (*p* < 0.01). Second, the correlation between each item and the total score was analyzed. The results showed that the correlation coefficients between the 23 items and the total score were 0.42–0.74 (p < 0.01). Third, the correlations between each item and its dimension were explored, and the correlation coefficients were 0.70–0.92 (*p* < 0.01). Finally, the correlation coefficients between each dimension and the total score were 0.73–0.79 (*p* < 0.01, *N* = 794).

#### Factor Analysis

An EFA was conducted on the 23 items to determine their underlying factor structure. The KMO value of sampling adequacy was 0.90, and Bartlett’s test for sphericity showed a significant difference (*p* < 0.001), which indicated that the data were appropriate for analysis (Tabachnick and Fidell, 2013). The final version of the MCAQ was composed of five factors and 21 items. After deletion, there were significant differences in the mean value of each factor between the high-score group and the low-score group. The cumulative variance contribution rate of the five factors was 72.17%. Furthermore, the items were internally consistent (Cronbach α = 0.89).

Factor 1 included six variables, i.e., management system and specification, management mode, professional pride, spirit of sacrifice, teamwork, and perseverance, reflecting the relationship between individuals and organizations, named organization and fusion ability. Factor 2 included five variables, i.e., interpersonal relationships, comrade-in-arms communication, superior and subordinate relationships, loneliness, and family communication, which reflected the relationships between individuals and others, named communication ability. Factor 3 included four variables: professional knowledge, learning ability, self-awareness, and command ability, reflecting the relationship between individuals and work, named learning ability. Factor 4 included four variables, i.e., work stress, environmental change, work frustration, and closed environment, reflecting the relationship between individuals and the work environment, named emotion regulation ability. Factor 5 included four variables, i.e., job change, secondary employment, career expectation, and emergencies, reflecting the relationship between individuals and their career, named vocational transformation ability. The factor loadings for these items are shown in Table 2.

**TABLE 2:** Factor loadings of the MCAQ.

Factor 1		Factor 2		Factor 3		Factor 4		Factor 5	
Item	Load	Item	Load	Item	Load	Item	Load	Item	Load
4	0.83	8	0.83	13	0.86	16	0.83	21	0.85
6	0.82	10	0.75	12	0.84	17	0.81	20	0.68
3	0.77	9	0.77	14	0.75	19	0.67	22	0.51
1	0.74	11	0.68			18	0.68	23	0.52
5	0.72								
2	0.65								

*MCAQ, Military Career Adaptability Questionnaire.*

### CFA

**FIGURE 1:**
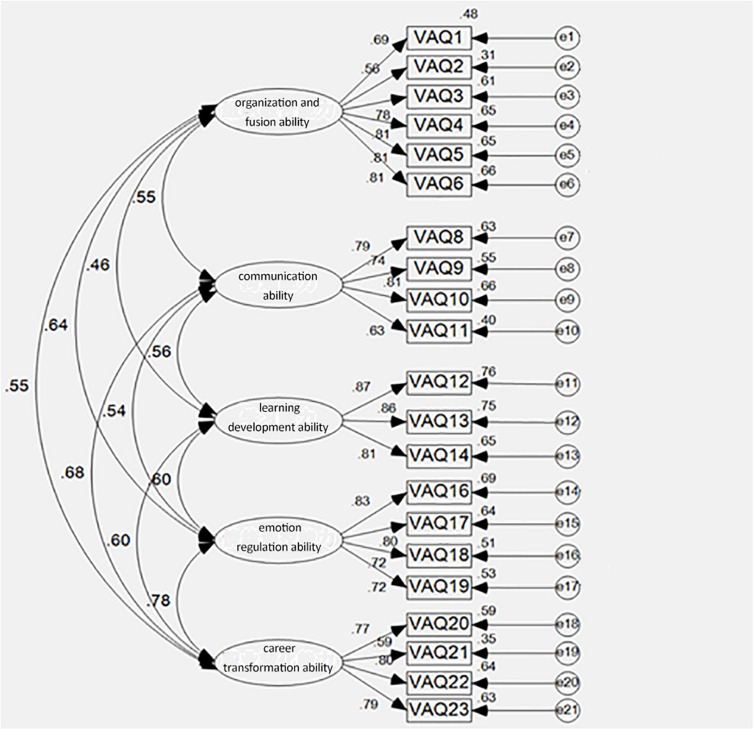
The standardized model of estimate values of the Military Career Adaptation Questionnaire.

To verify the structural validity of our questionnaire, the structural equation modeling statistical software AMOS 23.0 was used for CFA on the sample 2 data. Figure 1 presents a standardized model of the estimate values. The results showed that the normalized regression coefficients between all items and factors in the measurement model were 0.5–0.9 (*p* < 0.01). According to the results of EFA and the theoretical scheme, a structural model with 21 observed variables and 5 latent variables was established. The NFI, RFI, IFI, TLI, and CFI ranged from 0.87 to 0.91, *df* = 222, χ^2^/*df* = 3.39, GFI = 0.87, NFI = 0.88, CFI = 0.91, IFI = 0.91, TLI = 0.90, RMSEA = 0.07 <0.08. This analysis revealed that it was an adequate model that fit the data.

### Reliability and Validity Assessment

#### Reliability Test

The internal consistency reliability test of the MCAQ was conducted using the EFA of the samples. The effective sample size was 450. The results are presented in Table 3, showing that the MCAQ and its five dimensions had good internal consistency.

**TABLE 3:** Internal consistency reliability test of the MCAQ (*N* = 450).

Factor	Cronbach α	Test–retest reliability Cronbach α
Organization and fusion ability	0.89	0.88
Communication ability	0.83	0.82
Learning development ability	0.88	0.90
Emotion regulation ability	0.84	0.86
Career transformation ability	0.79	0.80
MCAQ	0.92	0.93

#### Criterion Validity

The criterion-related validity between the PCQ, JSQ, and MCAQ was analyzed based on the effective sample size, 474 participants, of the test sample. The total score of the MCAQ and the scores of its five dimensions were positively correlated with the total scores of the PCQ and JSQ (*p* < 0.01, two-sided) (Table 4). The test proved the beneficial criterion-related validity of the MCAQ.

**TABLE 4:** Correlations between the PCQ, JSQ, and MCAQ (*r*, *n* = 474).

	Organization and fusion ability	Communication ability	Learning development ability	Emotion regulation ability	Career transformation ability	MCAQ
Confidence	0.35**	0.50**	0.43**	0.37**	0.45**	0.53**
Hope	0.47**	0.54**	0.48**	0.52**	0.53**	0.65**
Resilience	0.40**	0.48**	0.45**	0.54**	0.54**	0.61**
Optimism	0.31**	0.36**	0.28**	0.33**	0.39**	0.43**
PCQ	0.46**	0.57**	0.49**	0.53**	0.58**	0.67**
JSQ	0.51**	0.42**	0.28**	0.43**	0.38**	0.54**

PCQ, Psychological Capital Questionnaire; JSQ, Job Satisfaction Questionnaire. **p < 0.01.

#### Structural Validity

Through correlation analysis, the factors of the MCAQ were highly correlated with the total score, and some factors were moderately correlated with each other (Table 5).

**TABLE 5:** Correlation matrix of various factors and total scores of the MCAQ (*N* = 474).

Factor	Organization and fusion ability	Communication ability	Learning development ability	Emotion regulation ability	Career transformation ability
**Organization and fusion ability**					
Communication ability	0.49**				
Learning development ability	0.45**	0.51**			
Emotion regulation ability	0.51**	0.51**.	0.52**		
Career transformation ability	0.39**	0.54**	0.48**	0.63**	
MCAQ	0.79**	0.77**	0.73**	0.82**	0.75**

**p < 0.01.

## Discussion

In this study, the EFA of the MCAQ showed that the statistical results were basically consistent with the theoretical hypothesis and that the five factors contributed 72.17% of the total variance. CFA revealed that the model had a high degree of structural validity, consistent with the assumptions regarding the empirical construction of military career adaptability. The results proved that this questionnaire can assess the career adaptability of military personnel. The reliability and validity analysis demonstrated that the dimensions of the questionnaire were highly correlated with the variables, indicating that the questionnaire has good reliability and validity.

### Empirical Construction of Military Career Adaptability

This study showed that a series of special abilities that individuals need to solve actual problems in their career is career adaptability. At present, the career adaptability of military personnel, who constitute a special occupational group, is not only related to the mental health and work efficiency of staff but also closely related to the fighting capacity of troops in the new era. Currently, the career adaptability of military personnel at all levels in different military units is evaluated by senior officers according to their experience. Therefore, a scientific and standardized scale cannot only help quickly and accurately investigate career adaptability but also detect strengths and weaknesses in adaptation to promote the career development of military personnel.

In quantitative research, the clear definition of a concept is the basis for operationalizing the concept and for quantitative research. This study focused on the actual problems faced by military personnel in their career development, adopted a “realistic” perspective for empirical construction, analyzed the main problems in the career development of military personnel and their ability to solve those problems, and gradually constructed the empirical dimensions of military career adaptability through logical methods such as generalization and induction. However, our study was based on an empirical construction, aiming to solve the actual problems faced by military personnel.

Compared with the career adaptability theories proposed by Savickas and Porfeli (2012) and Zhao et al. (2015), our study had a different temporal orientation of adaptability. As the core concept of career development theory, both Zhao and Savickas focused on future career planning and the psychological resources to cope with changes in the future. However, as a vocational group with a unified management mode and with absolute obedience, military personnel seem to have a relatively unified development path, while the ability to solve real problems is even more important because they will encounter more problems in career development. Military career adaptability is not a general concept referring to psychological resources or quality but a special ability required to meet the professional requirements of being a soldier. It is an actual ability that can be shaped and measured to effectively solve actual problems in the careers of military personnel.

Item screening is the key link in the development of the MCAQ. To improve the reliability and validity of the questionnaire, we analyzed each item in the questionnaire through item analysis, detected the differences between the high-score group and the low-score group, and screened out the high-quality items to constitute a questionnaire. In addition to expert evaluations, the correlation coefficients between each item of the questionnaire and the total questionnaire reached the level of statistical significance. The correlation coefficients between each item and the total score were 0.42–0.74 (*p* < 0.01), and the questionnaire had good content validity, suggesting good reliability and validity. This study showed that the MCAQ had high discriminant validity.

### Dimensions of Military Career Adaptability and Their Relationship

Military career adaptability includes five aspects, i.e., organization and fusion ability, communication ability, learning development ability, emotion regulation ability, and career transformation ability, corresponding to the relationships between military personnel and troops, military personnel and their comrades and relatives, military personnel and work, military personnel and stress scenarios, and military personnel and career opportunities, which are relatively independent and interrelated parts that construct the career adaptability of military personnel.

Among them, the organization and fusion ability dimension involves the relationship between individuals and organizations, i.e., the relationship between military personnel and troops, mainly to solve problems in the management of military units, as well as the matching of personality traits and work features. Communication ability involves the relationship between individuals and others, mainly to solve communication problems between officers and soldiers, especially in regard to superior–subordinate relations, and communication among comrades-in-arms and family members. Studies show that the influence of family was discerned as an important theme in career adaptability (Jennifer et al., 2016). Learning development ability involves the relationship between individuals and work, mainly to solve problems related to the knowledge and skills needed to accomplish tasks and to achieve career development, including military skills and professional technology. Emotion regulation ability involves the relationship between individuals and the work environment, mainly to solve stress-related problems at work, including environmental stress during military operations and work pressure. This dimension is a psychological experience index of military career adaptability. Career transformation ability involves the relationship between individuals and career opportunities, mainly to solve problems caused by career transformation in different positions.

Among the five dimensions of military career adaptability, organization and fusion ability, communication ability, learning development ability, and emotion regulation ability are the horizontal dimensions, while career transformation ability is the vertical dimension. Therefore, the structure of the questionnaire in this study is relatively complete.

The results of factor analysis provide a basis for determining the relative significance of each dimension in military career adaptability. According to the characteristic value of each factor, differences in career adaptability are mainly attributed to organization and fusion ability, suggesting that for military personnel with different characteristics, the greatest difference lies in organization and fusion ability, followed by emotion regulation ability, communication ability, career transformation ability, and learning and development ability. These results demonstrate that problems in reality are the main challenge for the career adaptation of military personnel, while improvement of military skills and professional technology is the most adaptable part in their careers.

### Reliability and Validity of the MCAQ

This study mainly used criterion-related validity and structural validity to reflect the overall validity of the questionnaire.

CFA showed that the five-factor structural model of military career adaptability obtained by EFA had good fit indices, moderate correlations among the factors, and high correlations between each factor and the questionnaire, indicating that the questionnaire had good structural validity.

For criterion-related validity, this study set the PCQ as the criterion. Psychological capital is a tool for transforming potential ability into actual ability (Jiang and Zhao, 2007). Psychological capital plays a positive role in the workplace and private life of individuals, and it can improve job satisfaction, reduce the tendency to resign, enhance commitment to the organization, reduce work pressure, and promote vocational well-being (Wu et al., 2012). Military personnel with strong career adaptability also have strong feelings of happiness, suggesting a certain correlation between psychological capital and career adaptability. The JSQ refers to survey respondents’ positive attitudes and emotions toward their work and related aspects; job satisfaction plays an important role in the job performance and subjective well-being of workers (Judge et al., 2001). The job satisfaction of soldiers has serious effects on morale and combat effectiveness. It is an important factor related to military career adaptability. According to Table 4, the dimensions of military career adaptability were significantly correlated with the JSQ and the dimensions of self-efficacy, optimism, hope, and resilience in the PCQ, indicating good criterion-related validity between military career adaptability, job satisfaction, and psychological capital.

This study mainly used the Cronbach α coefficient to measure internal consistency, and the results showed that the questionnaire had good internal consistency reliability. Our study also had good test–retest reliability, reflecting the high stability of the questionnaire.

## Conclusion

In conclusion, the MCAQ developed in this study has good reliability and validity and is consistent with psychometric requirements and the actual psychological development of military personnel. There were 21 items in the MCAQ, including five factors: organization and fusion ability, communication ability, learning development ability, emotion regulation ability, and career transformation ability. The MCAQ can be used as an effective tool to measure the career adaptability of military personnel.

## Data Availability Statement

The datasets generated for this study are available on request to the corresponding author.

## Ethics Statement

The studies involving human participants were reviewed and approved by Ethics Committee of Third Military Medical University. The patients/participants provided their written informed consent to participate in this study.

## Author Contributions

WZ was responsible for the original conception, data collection, and data analysis and made a major contribution to writing the manuscript. ML, LP, and YY constructed the original concepts and assisted in data collection. YX and CB assisted in data analysis and the study design and assisted in the drafting process. ML and YL critically revised the manuscript. All authors approved the final version of the manuscript for submission.

## Conflict of Interest

The authors declare that the research was conducted in the absence of any commercial or financial relationships that could be construed as a potential conflict of interest.

## References

[B1] Bates M. J. Stephen B. Jon H. Charlene S. Evette P. Monique M. (2010). Psychological Fitness. *Mil. Med.* 175 21–38. 10.7205/MILMED-D-10-0007320108838

[B2] Brown A. Bimrose J. Barnes S. Hughes D. (2012). The role of career adaptabilities for mid-career changers. *J. Vocat. Behav.* 80 754–761. 10.1016/j.jvb.2012.01.003

[B3] Gao X. Xin X. Zhou W. Jepsen D. M. (2019). Combine your “will” and “able”: career adaptability’s influence on performance. *Front. Psychol.* 9:2695. 10.3389/fpsyg.2018.02695 30723445 PMC6349723

[B4] Guan Y. Chen S. X. Levin N. Bond M. H. Luo N. Xu J. (2015a). Differences in career decision-making profiles between American and Chinese university students: the relative strength of mediating mechanisms across cultures. *J. Cross Cult. Psychol.* 46 856–872. 10.1177/0022022115585874

[B5] Guan Y. Zhou W. Ye L. Jiang P. Zhou Y. (2015b). Perceived organizational career management and career adaptability as predictors of success and turnover intention among Chinese employees. *J. Vocat. Behav.* 88 230–237. 10.1016/j.jvb.2015.04.002

[B6] Hartung P. J. Porfeli E. J. Vondracek F. W. (2011). Career adaptability in childhood. *Career Dev. Q.* 57 63–74. 10.1002/j.2161-0045.2008.tb00166.x

[B7] Hartung P. J. Taber B. J. (2008). Career construction and subjective well-being. *J. Career Assess.* 16 75–85. 10.1177/1069072707305772

[B8] Hu F. Zhao M. X. Lai W. Xia F. Huang Y. Feng Z. Z. (2018). Compilation and reliability & validity analysis of mental health ability questionnaire for army-men. *J. Third Mil. Med. Univ.* 6 466–472. 10.16016/j.1000-5404.201710167

[B9] Hu X. L. Cheng Y. (2017). Prospective personality, career adaptability and employee career growth: the moderating effect of job autonomy. *Hum. Resour. Dev. China* 2 16–23. 10.3969/j.issn.1004-4124.2017.02.003

[B10] Jennifer L. Peter M. Harsha N. P. (2016). A thematic analysis of career adaptability in retirees who return to work. *Front. Psychol.* 7:193. 10.3389/fpsyg.2016.00193 26925014 PMC4756173

[B11] Jiang J. W. Zhao S. M. (2007). Psychological capital and strategic human resource management. *Econ. Manag.* 9 55–58.

[B12] Jiang Z. (2016). The relationship between career adaptability and job content plateau: the mediating roles of fit perceptions. *J. Vocat. Behavi.* 95-96 1–10. 10.1016/j.jvb.2016.06.001

[B13] Judge T. A. Thoresen C. J. Bono J. E. Patton G. K. (2001). The job satisfaction-job performance relationship: a qualitative and quantitative review. *Psychol. Bulle.* 127 376–407. 10.1037/0033-2909.127.3.376 11393302

[B14] Luthans F. Luthans K. W. Luthans B. C. (2004). Positive psychological capital: beyond human and social capital. *Bus. Horizons* 47 45–50. 10.1016/j.orgdyn.2004.01.003

[B15] Luthans F. Youssef C. M. (2004). Human, social and now positive psychological capital management: investing in people for competitive advantage. *Organ. Dyn.* 33 143–160. 10.1016/j.bushor.2003.11.007

[B16] Maggiori C. Johnston C. S. Krings F. Massoudi K. Rossier J. M. (2013). The role of career adaptability and work conditions on general and professional well-being. *J. Vocat.Behav.* 83 437–449. 10.1016/j.jvb.2013.07.001

[B17] Negru-Subtirica O. Pop E. I. (2016). Longitudinal links between career adaptability and academic achievement in adolescence. *J.Vocat.Behav.* 93 163–170. 10.1016/j.jvb.2016.02.006

[B18] Nilforooshan P. Salimi S. (2016). Career adaptability as a mediator between personality and career engagement. *J. Vocat. Behav.* 94 1–10. 10.1016/j.jvb.2016.02.010

[B19] Pouyaud J. Vignoli E. Dosnon O. Lallemand N. L. (2012). Career adapt-abilities scale-France form: psychometric properties and relationships to anxiety and motivation. *J. Vocat. Behav.* 80 692–697. 10.1016/j.jvb.2012.01.021

[B20] Rudolph C. W. Lavigne K. N. Zacher H. (2017). Career adaptability: a meta-analysis of relationships with measures of adaptivity, adapting responses, and adaptation results. *J. Vocat. Behav.* 98 17–34. 10.1016/j.jvb.2016.09.002

[B21] Savickas M. L. Porfeli E. J. (2012). Career adapt-abilities scale: construction, reliability, and measurement equivalence across 13 countries. *J. Vocat. Behav.* 80 661–673. 10.1016/j.jvb.2012.01.011

[B22] Tabachnick B. G. Fidell L. S. (2013). *Using Multivariate Statistics*, 6th Edn Boston, MA: Pearson Education.

[B23] Wang Y. F. (2014). *Occupational Adaptability of Enterprise Employees: Measurement and Influence Mechanism.* Doctoral dissertation, Southwest University, Chongqing.

[B24] Worthington R. L. Whittaker T. A. (2006). scale development research: a content analysis and recommendations for best practices. *Couns. Psychol.* 34 806–838. 10.1177/0011000006288127

[B25] Wu W. T. Liu Y. Lu H. Xie X. X. (2012). Relationship between native psychological capital and occupational happiness. *Acta Psychol. Sin.* 44 1349–1370. 10.3724/SP.J.1041.2012.01349

[B26] Xie B. Xia M. Xin X. Zhou W. (2016). Linking calling to work engagement and subjective career success: the perspective of career construction theory. *J. Vocat. Behav.* 94 70–78. 10.1016/j.jvb.2016.02.011

[B27] Xu H. Liu T. Chen Y. W. (2017). “Research on the influence of employees’ career adaptability on occupational success,” in *2017 IEEE International Conference on Industrial Engineering and Engineering Management (IEEM)*, Piscataway, NJ: IEEE.

[B28] Zacher Hannes (2014). Career adaptability predicts subjective career success above and beyond personality traits and core self-evaluations. *J. Vocat. Behav.* 84 21–30. 10.1016/j.jvb.2013.10.002

[B29] Zhao M. X. Chen B. Luo Q. Feng Z. Z. (2018). Organizational affective commitment mediates effect of military transformational leadership in promoting psychological fitness. *J. Third Mi. Med. Univ.* 8 733–738. 10.16016/j.1000-5404.201711173

[B30] Zhao X. Y. Tan D. Y. Guo C. (2015). Development of the college students’career adaptability questionnaire. *J. Chin. Ment. Health* 29 463–469. 10.3969/j.issn.1000-6729.2015.06.012

